# Real-World Evidence on the Sensitivity of Preoperative Ultrasound in Evaluating Central Lymph Node Metastasis of Papillary Thyroid Carcinoma

**DOI:** 10.3389/fendo.2022.865911

**Published:** 2022-06-09

**Authors:** Fan Yao, Zhongyuan Yang, Yixuan Li, Weichao Chen, Tong Wu, Jin Peng, Zan Jiao, Ankui Yang

**Affiliations:** ^1^ Department of Head and Neck, Sun Yat-sen University Cancer Center (SYSUCC), Guangzhou, China; ^2^ State Key Laboratory of Oncology in South China, Sun Yat-sen University Cancer Center (SYSUCC), Guanghzou, China; ^3^ Collaborative Innovation Center for Cancer Medicine, Sun Yat-Sen University Cancer Center, Guangzhou, China

**Keywords:** papillary thyroid carcinmona, central lymph mode metastasis, real-world data, sensitivity, ultrasound

## Abstract

**Introduction:**

Guidelines for prophylactic dissection in clinical central negative node (cN0) of papillary thyroid carcinoma vary among different countries due to the uncertainty on the benefit of dissection. The Chinese guidelines recommend prophylactic central compartment lymph node dissection (pCLND) under professional technology. Preoperative ultrasound (US) evaluation of central lymph node determines the surgical strategy used. Sensitivity differs significantly when US is conducted by different physicians even in diverse hospitals. In this study, the aim was to explore why the Chinese guidelines were different from the America Thyroid Association (ATA) guidelines through the real-world evidence on the preoperative diagnosis of cN0.

**Methods:**

Preoperative US and surgical pathology data for 1,015 patients with PTC attending 13 Grade-A tertiary hospitals in 2017 were collected. A retrospective analysis using US assessment of CLNM was the conducted to explore the benefits of this approach in China. US physicians in our hospital were trained on scanning the thyroid gland and its regional lymph nodes in normalization. Data of 1,776 patients were collected under the same condition from 2012 to 2017, whose ultrasonography was performed by diverse physicians and doctors. Further, data of 339 patients evaluated by the same sonographer and operated by the same surgical team was collected between 2015 and 2017. In this set of data, US combined CT versus US alone was compared. Patients were grouped into metastasis group and non-metastasis group based on postoperative pathological diagnosis of CLNM. Diagnostic efficacy of US was evaluated.

**Results:**

A total of 925 patients who underwent preoperative ultrasonography in central lymph node, including 825 cases who underwent thyroidectomy and central lymph node dissection were included in this study. The sensitivity of ultrasonography in detecting CLNM was 23.18%, with occult metastasis rate of 40.8%. Data for 1,776 patients comprising paired ultrasonic report and pathological report were collected in our hospital, whose physicians underwent standardized training. The sensitivity was 37.58%. Furthermore, specialized evaluation showed high sensitivity in US/CT (84.58%) than US (58.21%) alone.

**Conclusion:**

Although the sensitivity of US could be enhanced by standardized training and combination with CT, the prevalence of low sensitivity of US in real-world multicenter data and the high occult metastasis rate indicated that the Chinese guidelines were based on the current conditions.

## Introduction

Incidence of thyroid carcinoma has significantly increased in the past few decades and is ranked fifth most common cancer type in females worldwide (1). In China, a 20% increase in thyroid carcinoma has been reported yearly (2). Papillary thyroid carcinoma (PTC) is the most common histological subtype accounting for approximately 80% of thyroid carcinoma cases (3). PTC is characterized by regional lymph node metastasis (LNM) at early stages. Most patients with PTC present with cervical LNM at the time of diagnosis (4). Central lymph node (CLN) is characterized by high rates of metastasis with CLN metastasis (CLNM) rate ranging from 20% to 90% (4).

The central compartment lymph node dissection (CCND) is recommended for diagnosis of clinical CLNM (cN1a) patients. However, guidelines on prophylactic central compartment lymph node dissection (pCLND) in patients with PTC with clinical negative node (cN0) vary among different countries due to varying reports on benefits associated with pCLND (5–12). This is attributed to lack of reliable large-scale prospective study due to the favorable prognosis of PTC. According to the ATA guidelines, patients with clinical central negative node (cN0) with low risk level are not recommended to perform pCLND (13). In China, the consensus proposed that pCLND should only be performed in low-risk patients in the case of effective preservation of parathyroid and recurrent laryngeal nerve (14, 15). This gave us curiosity to figuring out how the differences between the two guidelines came about. We tried to discuss it from the diagnosis of cN0, which determines the surgical strategy preoperatively. In addition, CLN occult metastasis rate of patients with cN0 could be as high as 84.3% (16), which changed tumor stage and postoperative management.

ATA recommended preoperative ultrasound (US) as the preferred evaluation method for thyroid nodules and cervical lymph nodes of thyroid malignance (13). Although preoperative US is highly effective in diagnosis of lateral lymph node metastasis (LLNM) with a sensitivity of 64%–93.8%, sensitivity of CLNM diagnosis is relatively low (10%–63%) and differs significantly in diverse centers (5–12). Preoperative evaluation of CLNM determines the surgical strategy; therefore, high sensitivity and accuracy of US should be ensured. Experience of sonographers, subjectivity, and messy US report format may all contribute to low sensitivity. In this study, we investigated the status of preoperative US in evaluating CLNM from 13 Grade-A tertiary hospitals in China and that in our single center. The findings of this study provide real-world evidence to help understanding how the ATA guidelines and the Chinese guidelines differ in management of cN0.

## Materials and Methods

### Ethics Statement

The studies involving human participants were reviewed and approved by the Ethics Committee of Sun Yat-sen University Cancer Center. All participants were informed of the purpose and risks of the study before signing written informed consents.

### Patients

Every 50–100 consecutive patients in 2017 with a diagnosis of papillary thyroid carcinoma from 13 Grade-A tertiary hospitals in China were enrolled in this study. Hospitals included in this study are located in different districts in China. Preoperative ultrasonography reports and pathology reports that were unmatched were removed and a total of 1,015 patients were enrolled in the study. Among the participants, 226 were male and 789 were female patients with a median age of 43.1 years (range from 9 to 76 years). Out of the 1,015 patients, 825 cases underwent CLN dissection confirmed by postoperative pathology.

A total of 1,776 cases with CLND in Sun Yat-sen University Cancer Center from 2012–2017 were collected, whose ultrasonography was performed by diverse physicians and doctors. In addition, a set of published data comprising 339 patients who were evaluated by the same sonographers and radiologists and operated by the same surgical team from June 2015 to December 2017 was retrieved. These data were referred as specialized evaluation, including US combined with CT. Specialized evaluation diminished the impact caused by subjective differences between different examiners and surgeons.

### Lymph Node Diagnostic Criteria

All materials were analyzed following guidelines from written reports of ultrasonography and postoperative pathology. Patients who underwent CLN dissection were grouped into metastasis group and non-metastasis group based on postoperative pathological diagnosis of CLNM. The diagnosis criteria was based on the fact that US report detected typical CLNM characteristics including focal hyperecho, microcalcification, peripheral blood flow signals, and round lymph nodes. For CT, CLNM would be diagnosed when one of the following features come up: gross calcification, uneven enhancement, short diameter greater than 5 mm, and focal cystic degeneration.

### Statistical Analyses

All statistical analyses were performed using SPSS software (version 23.0; IBM Corp, USA). The ratio of the cases that US reported as CLN to the cases that US did evaluation on CLN was referred as the detection rate. Sensitivity, specificity, accuracy, positive predictive value (PPV), and negative predictive value (NPV) of US and US/CT for estimating CLNM were analyzed using postoperative report as the golden standard. Weighted Youden index was used to estimate the diagnostic value of US and US/CT in screening suspicious CLNM. The formula of Jω was 2 (ω × sensitivity + (1 − ω) × specificity) − 1 (0 ≤ ω ≤ 1) (17).

## Results

### Current Condition in China

Data collected were collected from 1,015 patients attending 13 Grade-A tertiary hospitals in China (**Table 1**). A total of 925 patients underwent preoperative ultrasonography on CLN, whereas 825 patients underwent CLN dissection. In addition, 202 cases underwent lateral lymph node dissection synchronously. The participants comprised 226 male and 789 female patients with a median age of 43.1 years (range from 9 to 76 years).

**Table 1:** The basic situation of preoperative US evaluation in13 Grade-A tertiary hospitals among China.

Hospitals	Cases	LN-Evaluated	US-diagnosed CLNM (Strict Criteria)	CLND	No CLND with CLNM (Strict Criteria)	Pathological CLNM	Pathological CLNM With LLNM	CLND with No US-Diagnosed CLNM	Occult CLNM
**Hospital A**	100	100(100.0%)	2 (2.0%)	56	1	19	0	55	18(32.7%)
**Hospital B**	67	67(100.0%)	0 (0%)	67	0	29	19	67	29(43.3%)
**Hospital C**	79	67 (84.8%)	0 (0%)	77	0	34	14	77	34(44.2%)
**Hospital D**	72	53 (73.6%)	1 (1.4%)	70	0	35	7	69	34(49.3%)
**Hospital E**	99	92 (92.9%)	4 (4.0%)	99	0	28	5	94	24(25.5%)
**Hospital F**	106	105(99.1%)	35 (33.0%)	102	0	59	25	61	25(41.0%)
**Hospital G**	50	50(100.0%)	2 (4.0%)	50	0	20	5	44	15(34.1%)
**Hospital H**	21	15 (71.4%)	0 (0%)	18	0	6	0	18	6(33.3%)
**Hospital I**	74	73 (98.6%)	8 (10.8%)	74	0	40	9	64	31(48.4%)
**Hospital J**	97	97 (99.0%)	15 (15.3%)	96	1	47	11	79	31(39.2%)
**Hospital K**	99	97 (98.0%)	1 (1.0%)	24	0	15	2	18	10(55.6%)
**Hospital L**	98	98(100.0%)	15 (15.3%)	41	3	29	12	26	15(57.7%)
**Hospital M**	53	11 (20.8%)	0 (0%)	51	0	23	6	51	23(45.1%)
**Total**	1,015	925(91.1%)	83 (8.2%)	825	5	384	115	723	295(40.8%)

Only one center out of the 13 Grade-A tertiary hospitals showed low assessment on CLN (20.8%), whereas other hospitals performed CLN evaluation on most cases (71.4%–100.0%) (**Table 1**). Notably, four hospitals performed CLN evaluation on all patients.

The sensitivity, specificity, accuracy, PPV, and NPV of US were 23.18%, 97.05%, 62.67%, 87.25%, and 59.2% (**Table 3**).

Surgical strategies determined by preoperative US varied from hospital to hospital. Ten hospitals performed prophylactic CLN dissection (pCLND) regardless of detection of CLNM using US. Hospital A, K, and L did not carry out pCLND if US report showed no CLNM. In addition, these hospitals did not perform CLND even when US reports showed CLNM in a few cases. CLNM rate was 46.5% (28.3%–70.7%), whereas the LLNM rate was 13.9% (0.0%–29.3%) (**Table 1**). Hospitals with higher CLNM rate showed higher LLNM and higher detection rate such as hospital F and L. For those without CLNM, the occult metastasis was generally high (25.5%–57.7%) (**Table 1**).

These findings show differences in evaluation level of ultrasonography among different hospitals. Sensitivity and diagnostic value of preoperative were low and varied greatly. In addition, surgical strategies guided by ultrasonography for CLND differed among hospitals. These factors contributed to the high rate of missed CLNM.

### Standardization of US Improves Sensitivity of US

Experience and subjectivity of sonographers affects the sensitivity of US. In our hospital, US physicians underwent unified training on thyroid and its regional lymph nodes. The training included scanning of CLN based on subregions to prevent omission, extending the scanning range by adopting flexible methods such as longitudinal scanning with probe on areas that are difficult to detect. Furthermore, they were required to use a normalized US report format to minimize subjectivity of sonographers.

A total of 1,776 cases were reported (**Table 2**). The sensitivity was higher than the Multicenter Evaluation (37.58% vs. 23.18%) (**Table 3**). In addition, the specificity, accuracy, PPV, and NPV of US were 91.94%, 61.88%, 85.22%, and 54.36%, respectively (**Table 3**).

**Table 2:** The basic situation of preoperative US evaluation in our single medical center.

		Cases	LN-Evaluated	US-Diagnosed CLNM	CLND	Pathological CLNM	Pathological CLNM with LLNM
Standardized Training^a^		1,776	1,776 (100.0%)	433 (24.4%)	1,776	982 (55.29%)	392
Specialized Evaluation^b^	US	339	339 (100.0%)	136 (40.1%)	339	201 (59.29%)	/
	CT/US	339	339 (100.0%)	210 (61.9%)	339	201 (59.29%)	/

a Standardized training was performed by different physicians and surgeons.

b Specialized evaluation was performed by the same group of physicians and surgeons.

**Table 3:** The diagnostic value of preoperative US in CLNM.

			Pathological Diagnosis		Sensitivity(%)	Specificity(%)	Accuracy(%)	PPV(%)	NPV(%)	Jω (ω = 0.6)
		Ultrasonic Diagnosis	CLN positive	CLN negative						
**Multicenter Evaluation**	Multicenter	CLN positive	89	13	23.18%	97.05%	62.67%	87.25%	59.20%	0.05456
		CLN negative	295	428						
Standardized Training^a^		CLN positive	369	64	37.58%	91.94%	61.88%	85.22%	54.36%	0.18568
		CLN negative	613	730						
Specialized Evaluation^b^	US	CLN positive	117	19	58.21%	86.23%	69.62%	86.03%	58.62%	0.38836
		CLN negative	84	119						
	US/CT	CLN positive	170	40	84.58%	71.01%	79.06%	80.95%	75.97%	0.58304
		CLN negative	31	98						

a Standardized training was performed by different physicians and surgeons.

b Specialized evaluation was performed by the same group of physicians and surgeons.

Despite that the standardized training might reduce the difference caused by subjectivity, lack of experience on determination of CLNM and differences in diverse surgical teams also had effect on the sensitivity of US. Therefore, 339 patients evaluated by the same sonographers and radiologists and operated by our surgical team between June 2015 and December 2017 were recruited in the study. In addition, the diagnostic value of US combined with CT (US/CT) was also evaluated for these patients.

CLNM rate was similar to the standardized training (59.29% vs. 55.29%) (**Table 2**). Sensitivity, specificity, accuracy, PPV and NPV of US alone were 58.21%, 86.23%, 69.62%, 86.01%, and 58.62%, respectively (**Table 3**). Sensitivity, specificity, accuracy, PPV, and NPV of US/CT were 84.58%, 71.01%, 79.06%, 80.95%, and 75.97%, respectively (**Table 3**).

Increased sensitivity is important for success of surgeries. Therefore, the weighted Youden Index (Jω) was calculated with setting ω as 0.6 to estimate the diagnostic value of US in CLNM sensitivity. Jω of Multicenter Evaluation was 0.06, and data from our hospital could be increased to 0.19. Besides, Jω of US/CT was higher compared with that for US alone (0.58 vs. 0.39) (**Table 3**).

## Discussion

CLNM rate in patients with PTC is approximately 90%, whereas occult metastasis rate ranges between 30% and 84% (16). Studies report contradicting results about CLND associated with PTC, mainly the appropriate timing for performing pCLND in patients with cN0. The ATA guidelines provide limited information on pCLND. These guidelines recommend that surgery on pCLND should be performed for high-risk patients such as T3–T4 stages and extrathyroidal extension (ETE) and pCLND should not be performed on low-risk patients like patients with T1–T2 stages (13). Guidelines in most countries are similar to the ATA guidelines (11, 12). On the contrary, the Japanese Society of Thyroid Surgeons/Japanese Association of Endocrine Surgeons recommends routine pCLND to reduce severe complications of surgery after recurrence, which is possibly related to low rates of radioactive iodine (RAI) treatment in Japan (10). Although these guidelines have some differences, decision-making should be balanced between risks and benefit based on experience of the surgeons.

The pros and cons of pCLND have not been fully explored. This is mainly because most studies on pCLND are retrospective studies with poor evidence. Previous studies report that pCLND reduces recurrence rate and improves survival rate in patients with high occult CLNM (18). Hospitals who perform prophylactic dissection of cN0 report that the rate of occult metastasis of lymph nodes is high in the central area. Preventive dissection reduces recurrence rate and improves survival rate of patients (18). However, hospitals that do not carry out preventive dissection avoid the procedure to minimize postoperative complications and believe that there is no scientific evidence on the role of pCLND in improving survival of patients (19). Papillary thyroid carcinoma is associated with advanced prognosis, and the longer follow-up time hinders observation of the outcome of preventive dissection. A few studies have report the 10-year OS of patients with pCLND, with significant variation in results (18–20). Most included patients who underwent pCLND also received radioactive I131 therapy (RAI) when they found lymph node metastasis after surgery, thus explaining these differences (18). The average dose of RAI was higher compared with those who did not undergo pCLND, and these patients had strict control standards in TSH suppression treatment. RAI and TSH suppression treatments partially substitute pCLND. Several studies report that patients with pCLND have a higher rate of temporary hypocalcemia and temporary recurrent laryngeal nerve injury. However, no significant difference in incidence of permanent hypoparathyroidism and permanent recurrent laryngeal nerve injury (21). In addition, previous studies did not consider development and changes of treatment strategies and differences in the level of experience of surgeons and have shorter follow-up time, and only few multicenter studies have been carried out. Notably, conducting a large-scale prospective study is challenging. ATA predicted that a prospective multicenter randomized controlled study of pCLND would take 7 years with approximately 5,840 patients, and it would 20 million dollars (22). Recently, Ahn et al. published a prospective randomized controlled trial to assess the efficacy of pCLND. The results found that although patients with pCLND showed higher CLNM rate, there was no difference between the local recurrence and postoperative complications, which made them think that patients with cN0 may not need pCLND (23). The study provides strong evidence for the absence of pCLND in patients with cN0, but we still need to consider that a 50-month follow-up face may not fully predict the long-term risk pf PTC, whose 10-year OS could be as high as 90% after a follow-up of 50 months or so.

The Chinese guideline for differentiated thyroid carcinoma recommends that pCLND in low-risk patients should only be performed under the technical guarantee of effective preservation of parathyroid and recurrent laryngeal nerve (14). In this multicenter study, some hospitals performed pCLND routinely, whereas others did not carry out routine pCLND (**Table 1**). These triggered our curiosity about the differences between ATA and Chinese guidelines in pCLND.

ATA guidelines indicate that US is the first-choice diagnostic approach for thyroid and its regional lymph nodes. US evaluation on CLNM affects the diagnosis of cN0 and surgical strategy. However, it is associated with low sensitivity ranging between 10% and 63%, when used for CLNM, due to the narrow area of the CLNs. The tumor is located deeper and lymph nodes in this area are small, thus affecting detection of microcalcification and cystic degeneration. In addition, it is difficult to display the color flow for the effect of air in the trachea and esophagus next to the lymph nodes. Despite these limitations, preoperative color Doppler US is the preferred assessment method in many hospitals across our country due to its ease of operation, lack of requirement of radiation, and ease of judgment between benign and malignant nodes. Surgeons mainly rely on preoperative US reports to assist in formulation of surgical strategies. Therefore, this study explored the current status of preoperative US evaluation in multicenters across China. Besides, we shared the US evaluation on CLNM at a single-center level. Those real-world evidence helped to figure out if the Chinese guideline was more suitable for the CLN management in China.

The findings of this study show an overall sensitivity of a multicenter is low. In our multicenter study, most hospitals would assess the lymph nodes in the central neck and report them in the US report, except that Hospital M had a lower assessment rate of 20.8% (11/52). However, the diagnosis rate of CLNM in the central area varied significantly. In Hospital K, although 98% patients underwent detection of lymph nodes, the diagnosis rate of malignant lymph nodes was 7% (7/97). Only 24% (24/99) of patients in Hospital K underwent CLND guided by preoperative US. The postoperative CLNM rate was 62.5% (15/24) and the occult metastasis rate was as high as 55.6% (10/18). Hospital F reported highest diagnosis rate of 38.7% (41/105), but it still reported a high occult metastasis rate (41%, 25/61). This difference can be attributed to the location of CLN, making it difficult to assess the features of typical metastatic CLNs. This challenge then results in failure to distinguish benign and malignant nodes. Most hospitals performed CLND for patients with cN0 evaluated by preoperative US, whereas a few hospitals routinely performed CLND with or without cN0. In addition, the overall occult metastasis rate was 40.8% (295/723). These results indicate that US evaluation on CLNM has poor accuracy rates in the real world, which brings high occult metastasis.

US examination is highly subjective, and experience of inspectors affects accuracy of preoperative US. Standardization of preoperative US evaluation in CLN can be improved by normalizing US report format, recording lymph node in detail, replacing subjective judgments by objective factors and conducting professional training to minimize individual differences. We observed that the US report format in the 13 hospitals was uneven, and the description of thyroid and lymph nodes was not complete and objective. Therefore, we investigated the data from our medical center, whose US physicians underwent unified training on thyroid and its regional lymph nodes and the normalized US report format. The result showed that the US on CLNM was a little more sensitive, but it was not high enough (**Table 3**). It is known that lacking of experience and difference in diverse surgical groups may hinder identification of typical CLNM. Further, a specialized evaluation was carried out by the same physicians and surgeries were performed by our surgical team. The sensitivity of CLNM significantly increased compared with those for the standardized training, which was performed by diverse groups of sonographers and surgeons. Combination of multiple preoperative methods can improve sensitivity of CLNM through advances in detection techniques (5, 24). Suh et al. reported that the sensitivity of CLNM was 38% when using US alone, whereas the sensitivity of CLNM after combining US and CT examination was 57% (25). CT and US are highly used in routine examination; therefore, we compared the sensitivity of combined US and CT with the sensitivity of US alone for specialized evaluation. Combination of the two approaches significantly increased the sensitivity of CLNM. In addition, there are several approaches to improve the sensitivity of CLNM. Artificial intelligence is widely used in medical field, mainly in medical imaging (26). AI can independently learn and identify various data in images to provide objective evaluation and minimize errors caused by subjectivity of physicians. Therefore, it is an important tool for improving medical imaging. Li et al. analyzed US imaging data of thyroid cancer by deep convolutional neural networks and reported similar sensitivity and better specificity compared with experienced radiologists in identification of malignant thyroid nodules (27). Lee et al. reported that CT combined with a computer-aided diagnosis system (CADS) based on deep learning accurately evaluates the features of cervical lymph nodes in thyroid cancer (28).

Our study shows that the accuracy of preoperative US in diagnosis of cN0 significantly varied among different hospitals in the country, which made the management of CLN different. Consistent with many reports, US/CT improved sensitivity of CLNM significantly in our medical center. However, considering that there are so many hospitals across China, whose level is uneven, and the large population, US is a more convenient, economical, and widely used tool. We tried to provide the real-world evidence on the sensitivity of US and share our single-center experience on improving sensitivity. The 13 hospitals in our study were located in different regions of China, and they all had good reputations in their respective regions. Even these excellent hospitals showed low sensitivity, we might discover why the Chinese guidelines shared diverse opinion on pCLND. It did take the diagnostic difficulty of cN0 into account.

Our study had a few limitations. First, although this was a multicenter study, it used retrospective analysis and the sample size was small. Second, the formats of US reports were not uniform and standardized, causing differences in detection of lymph nodes. Third, hospitals that were included in the multicenter study were Grade-A tertiary hospitals. The status for preoperative US in junior hospitals was not evaluated; thus, in the findings of this study, it does not reflect challenges in CLNM evaluation in these hospitals.

## Conclusion

The sensitivity of preoperative US for the diagnosis of CLNM are low and significantly vary among different hospitals, and sensitivity can be increased through standardized training and specialized evaluation. A combination of multiple examinations such as US/CT can improve preoperative evaluation. These findings reflect the real-world management of CLN in China. Considering the poor sensitivity of US, the Chinese guidelines in the treatment of cN0 were in line with reality.

## Data Availability Statement

The original contributions presented in the study are included in the article/**Supplementary Material**. Further inquiries can be directed to the corresponding author/s.

## Ethics Statement

The studies involving human participants were reviewed and approved by Ethics Committee of Sun Yat-sen University Cancer Center. Written informed consent to participate in this study was provided by the participants’ legal guardian/next of kin.

## Author Contributions

All authors listed have made a substantial, direct, and intellectual contribution to the work, and approved it for publication.

## Conflict of Interest

The authors declare that the research was conducted in the absence of any commercial or financial relationships that could be construed as a potential conflict of interest.

## Publisher’s Note

All claims expressed in this article are solely those of the authors and do not necessarily represent those of their affiliated organizations, or those of the publisher, the editors and the reviewers. Any product that may be evaluated in this article, or claim that may be made by its manufacturer, is not guaranteed or endorsed by the publisher.

## Supplementary Material

The Supplementary Material for this article can be found online at: https://www.frontiersin.org/articles/10.3389/fendo.2022.865911/full#supplementary-material
